# Leading a Digital Transformation in Pharmacy Education with a Pandemic as the Accelerant

**DOI:** 10.3390/pharmacy9010019

**Published:** 2021-01-12

**Authors:** Edith Mirzaian, Kari L. Franson

**Affiliations:** Titus Family Department of Clinical Pharmacy, University of Southern California School of Pharmacy, Los Angeles, CA 90033, USA; kfranson@usc.edu

**Keywords:** digital transformation, online education, educational innovation

## Abstract

The global COVID-19 pandemic has not only posed a challenge to education but created an opportunity to spearhead a digital transformation and the novel delivery of a Pharm.D. curriculum. The process to transform the curriculum in a sustainable and iterative manner involved multiple steps including: (1) Communication, (2) Maintaining faculty engagement, (3) Allowing outside the box thinking, (4) Providing resources and tools and (5) Creating accountability and timelines. At our institution, we have been interested in digital transformation since completing our interview of global leaders. We began our journey using the current COVID-19 pandemic as an accelerant for change. Digital transformation in any industry is not a simple undertaking. However, with planning, aligned organizational interests, consistent and regular communication, provision of resources and tools, engaging faculty and creating accountability and timelines with deliverables the implementation can be successful. When the global pandemic wanes and educational institutions commence in-person classes, having undergone the stages of digital transformation, we will be able to embrace these changes and transform education, not having to reproduce pre-pandemic educational systems.

## 1. Introduction

For many years, leaders of educational institutions have considered the best manner in which to take advantage of the technological tools enabling the realization of e-Learning. Over time, these deliberations identified several benefits and barriers which have been debated in the literature. Will e-Learning enable the realization of competency-based education? Will online education enhance access? Will online education be more or less affordable? Can professionals be socialized to the profession via distance approaches? Can or will current faculty learn to use these technologies? Is online learning comparable to that which is provided face-to-face? [1,2]. With the onset of the COVID-19 pandemic we have seen many school leaders be able to move past these barriers to digitize their curricula and provide their learners with distance-based education. However, it is important to recognize that digitization is not necessarily digital transformation and many of the identified barriers still exist. This paper will discuss the definition of digital transformation, highlight some of the recent literature regarding pharmacy education’s efforts to provide distance-based education during the recent COVID pandemic, review the common barriers to digital transformations across industries including education and describe our own institution’s efforts to lead a digital transformation of our curriculum at the onset of the COVID pandemic.

### 1.1. Understanding Digital Transformations

Learning at a distance via computer technology is not a new phenomenon. A few readers may recall the Electronic University Network established in 1983 to provide students with access to degree-bearing institutions without attending them [3]. Distance education using other technologies such as telephones, televisions and the postal service go back even further, with examples such as the University of London External Programme offering full degrees through distance learning since 1858 [4]. But it wasn’t until late 1997, when Elliott Masie said, “Online learning is the use of network technology to design, deliver, select, administer, and extend learning” [5] that leaders began to think about the way in which technology can extend and therefore transform education. While there is not one definition of digital transformation that is universally accepted, we appreciate the broad scope characterized by i-scoop “Digital transformation is the profound transformation of business and organizational activities, processes, competencies and models to fully leverage the changes and opportunities of a mix of digital technologies and their accelerating impact across society in a strategic and prioritized way, with present and future shifts in mind” [6]. It is also important to clarify what digital transformation is not. Taking a normally orally delivered lecture, recording it and converting it into a digital (i.e., computer-readable) format is digitization. The result is a digital representation, not a digital transformation. Leaders who embrace the broad definition of a digital transformation are more likely to increase agility, improve learner experiences, change educational business models, and reduce costs. Based upon interviews of global pharmacy leaders, this is precisely what pharmacy education has needed before and will need after the pandemic [7].

### 1.2. Academic Pharmacy’s Response to the Pandemic

**Figure 1:**
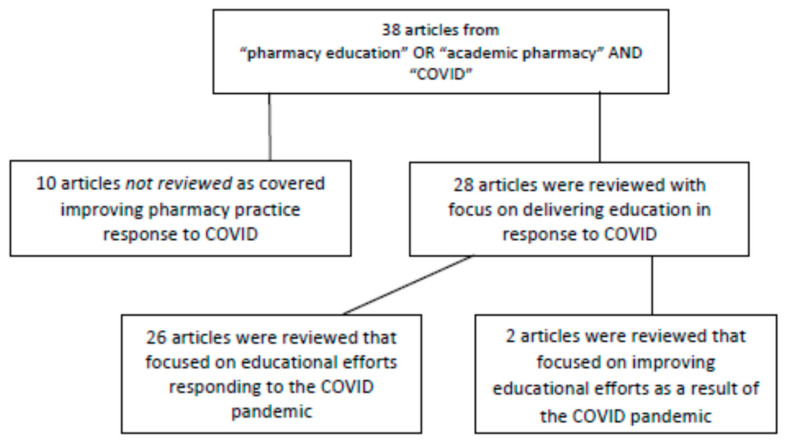
Literature review flow chart.

It has been less than a year since the start of the COVID-19 pandemic and a PubMed search of “pharmacy education” OR “academic pharmacy” AND “COVID” between 1 December 2019 and 1 November 2020 already identified 38 articles written about the global pharmacy academy response to the pandemic. Ten of the articles were about pharmacy practice and were not reviewed (Figure 1). Of the remaining 28 [8,9,10,11,12,13,14,15,16,17,18,19,20,21,22,23,24,25,26,27,28,29,30,31,32,33,34,35], most were solely focused on addressing the challenges presented by current events, with only a few describing seizing opportunities that represent the needs of the future. In “Pharmacy Education Crosses the Rubicon”, the authors stated that “the pre-COVID-19 global response of the Academy to [technological] changes around us might have been described as incremental” and “the pandemic has forced us into a state of out-of-the-box thinking and creative problem-solving” [24]. It is apparent from the evidence presented from the 26 published articles that our pharmacy academy has done a remarkable job responding to the pandemic. Many of the 26 articles describe approaches to sustain the current levels of education. Whereas two articles describe responding to the pandemic as new opportunities [9,18]. But were these changes inevitable as there was no alternative? What happens when the global health crisis wanes and the world looks to return to normal? Will the academy seek to reproduce the pre-pandemic educational systems or will we embrace these changes and transform education? Will the academy be strategic and try to transform our educational processes to accelerate the needed change? The academy should recognize that we have crossed the Rubicon and we need to embrace the opportunity presented to us and improve our educational processes.

### 1.3. Achieving Digital Transformation

It should be noted that achieving digital transformations are easier said than done. In 2019, Tabrizi et al. reported that across a wide range of industries, 70% of all digital transformation efforts did not reach their goals, resulting in hundreds of billions of dollars wasted [36]. Tabrizi’s number one rationale for failure was the lack of vision or purpose, specifically not communicating both a desire to improve “staff” efficiency and “customer” experience. Secondly, Tabrizi adds that there appears to be a fine line for bringing in subject matter experts. They need to balance their ability to lead, stimulate, engage and collaborate with existing staff without feeding the feeling of employment jeopardy and being replaced. Transformation projects that were seen as having a beginning and end also were more likely to fail than those that had a more iterative process. Lastly, many projects were simply abandoned due to lack of patience, funding or resources to see through to the end [36]. At the University of Southern California (USC) we have been interested in starting our journey toward digital transformation since completing our interview of global leaders in 2019. Unlike a business process or a product launch, this process does not have a beginning and an end, but rather is an iterative process that will undergo continued improvement and revision based on faculty and student feedback, data and overall process experiences and continue to evolve over time. This paper reports on the beginning of our journey toward digital transformation in which we used the current COVID-19 pandemic as an accelerant for change.

## 2. Methods

### 2.1. Responding to the Crisis

On 6 March 2020, the provost notified university faculty that within five days there would be a three-day (11–13 March) campus-wide pilot to test delivering virtual curricula. As school administrators responsible for academic affairs and curriculum, we proceeded to engage in planning discussions amongst leadership and faculty to launch a coordinated effort in developing these new educational delivery methods. Our respective experiences led us to move forward in a coordinated manner in order to ensure student success. At this point in the semester, our third-year students (P3) were completing their courses and preparing to start their fourth-year advanced pharmacy practice experiences (APPEs) or rotations the following week. The second-year (P2) students already completed their introductory pharmacy practice experiences (IPPEs), IPPE seminars and electives, and had two core courses remaining to complete, one of which was case conference conducted in small groups with resident facilitators and the other a classroom-based therapeutics course. The first-year (P1) students had five ongoing courses, three of which were being conducted in a flipped classroom model. During the first 3 virtual teaching days, we had to administer observed structured clinical exams (OSCEs) for two courses, a final exam, and lectures for several courses. Course coordinators who were familiar with Zoom^®^ conferencing technology partnered with other faculty coordinators to move the OSCEs onto the Zoom platform. It became clear that efficiencies were gained by moving several separately scheduled sessions into one session via Zoom rooms, with a “waiting room” and individual OSCE sessions proctored by faculty and graduate student teaching assistants. Many of the courses were coordinated by faculty who were already applying a variety of teaching methodologies such as flipped classroom instruction, team-based learning and small group discussions. These faculty also applied high-level use of instructional and conferencing technology, recording features in the learning management system, and Zoom virtual meeting software to change their teaching to the virtual environment.

Following those first 3 days, we were instructed to continue teaching virtually for two more weeks until further notice. At this point, we realized that communication and faculty engagement was key. The pandemic was going to require all faculty to adapt, but we were going to focus our efforts on identifying efficiencies and maintaining the quality educational experience. Weekly P1–P3 course coordinator meetings were held to provide faculty a collaborative forum to discuss course status, experiences, successes, and challenges. In these meetings, faculty shared the tools, technology and processes they used to deliver their courses. Additionally, information technology staff (IT) and school administrators were present to communicate that it was OK to start simply, as well as provide additional resources and solutions to address immediate challenges. This forum was critical to keep faculty engaged, address needs to continue teaching, reduce anxiety, share ideas, resources and solutions, and uphold faculty morale.

We also communicated with students understand their experiences with the change in learning. Students faced many challenges at home including, but not limited to, reliable internet connectivity, managing home life while attending live classes, and caring for dependents. We learned it was critical for us to provide flexible options and set realistic expectations regarding assignments and exam scheduling. Instructors were asked to record their presentations and lectures on the learning management system (LMS) for students to access in an asynchronous fashion. Discussion boards were enabled to address content questions as students studied the asynchronous material. Live, synchronous question and answer sessions were scheduled for each course to provide students a live encounter with course instructors and opportunities to clarify content.

Timed exams were administered via web-based examination software and made available from 8 am to 11 pm to accommodate students who needed flexibility in timing, internet availability and dedicated quiet space at home. Most exams were written as open book in order to reduce the level of anxiety and minimize concerns over compromises in academic integrity.

### 2.2. Committing to a Digital Transformation

The looming unknown state of the Fall semester meant we had to be prepared for an entirely virtual delivery of our curriculum. This was not just a challenge, but an opportunity for us to spearhead the implementation and novel delivery of a Pharm.D. curriculum. The technical transformation of our Spring courses to the virtual environment set the tone for the overall transformation of the curriculum in Fall. The successes were the result of multiple factors including a critical number of faculty who were adept with instructional technology and willing to collaborate with and support their fellow colleagues, regular communication with course coordinators and faculty to address teaching needs, the experimentation of various tools and resources, and communication with students with regard to the vision and purpose of changes and to iterate based on their feedback. Administratively we began to meet with Fall 2020 course coordinators in March 2020 to discuss conversion to online teaching, specific needs for courses and faculty, curriculum delivery models, course scheduling, standardizing and coordinating processes and support for assessments in a virtual environment. These meetings led us to believe that there was a real prospect to mobilize faculty to transform the entire didactic portion of the curriculum to an appropriately structured format for virtual education in a professional degree program. The process to carry out this vision over a three-month period involved the following:

(1) *Communicating*. The core principle that excellent teaching is not optional provided a shared goal amongst the faculty as a whole. Administration often communicated to both faculty and students that the pandemic required everyone to approach education differently and that pharmacy education was not going to return to “normal” any time soon.
(2) *Maintaining faculty engagement*. Fortunately, a critical mass of faculty colleagues was committed to working hard to ensure the continuity of high-standard and engaging course delivery. Frequent and regular communication through semester planning meetings, regular email reminders of meetings and resources, and consistent messaging from academic and faculty deans served as constant points of connection with the faculty as a group.
(3) *Allowing outside the box thinking*. Being a professional degree program, the USC School of Pharmacy is allowed exceptions to university-wide schedules and processes by university administration. Thus, the School of Pharmacy had a blank template in which to schedule courses. A traditional weekly class schedule primarily consisting of live lectures would not work in the virtual education format since that would lead to 6 to 8 h of instruction per day, four days a week of attending class on online platforms, causing screen fatigue and possible disengagement. Course coordinators worked with administration to create a completely new fall schedule consisting of asynchronous and synchronous didactic learning sessions as well as IPPEs and required live clinical skills training sessions. Each P1-P3 didactic course was allocated one 3-h synchronous session block either weekly or every other week. The synchronous sessions for each curriculum year were scheduled over two days of the week, with some fewer credit hour courses scheduled on alternating weeks. The remaining days of the week were dedicated to assessments, faculty office hours, question and answer sessions, completing asynchronous work, and one day per curriculum year for IPPEs. All knowledge level instructional content was delivered in the asynchronous format with the synchronous sessions designed as application-based learning sessions engaging students with the content expert faculty applying and reinforcing the content from the asynchronous material.
(4) *Providing Resources and Tools*. The alignment of administrative, departmental and faculty commitment to train, develop, and properly resource the process of digital transformation was critical to the success of this process. School administration supported the proposed process, technology, limited-contract experts to provide additional training and advisement, and faculty time dedicated to professional development and training in online course design. Faculty accepted many of the professional development options provided to them.
  (a) All School of Pharmacy faculty, across four departments, received strong encouragement from the Dean and his leadership team to attend the 6-week, Accelerated Summer Intensive for Online Teaching provided by the USC Center for Excellence in Teaching (USC CET).
  (b) An initial training session was offered in May 2020 to faculty. This 2-h session *Key Values for Online Learning and Backwards Design* and *Transforming Your Lecture and Avoiding the Bloat* was developed and presented by instructional designer experts who designed the online professional doctorate education at the University of Colorado Skaggs School of Pharmacy and Pharmaceutical Sciences (CUSSPPS).
  (c) In July 2020, the same group of experts from CUSSPPS offered training focused on *Team Teaching*, a practice that is common in our Pharm.D. curriculum.
  (d) Curriculum support staff, our USC CET Faculty Fellows from each of the four departments and the contracted CUSSPPS instructional designers worked with individual faculty to provide feedback on course design, learning objectives, aligning assessments with learning objectives, designing course modules complete with asynchronous and synchronous sessions, pre-work and post-work for each module, and assessments.
  (e) The USC CET designated one of their instructional designers to the School of Pharmacy to provide assistance to individual faculty who need further guidance and assistance.
  (f) Faculty were provided financial support from department chairs to attend the American Association of Colleges of Pharmacy Teachers’ Seminar during the Annual Virtual Meeting in July 2020.
  (g) A virtual course template was installed in the LMS to maintain a consistent organization of module material across all courses.
(5) *Creating accountability and timelines*. A timeline of course development including submission dates for work products were set for a 6-week period over June and July 2020. The work products were aligned with the assigned work products faculty were to develop as part of the USC CET Accelerated Online Teaching Intensive. The products faculty submitted were:
  (a) Course Alignment Grid with course learning objectives and aligned assessments
  (b) Course schedule with modules organized in the LMS. Faculty were to prepare asynchronous material, pre-work, synchronous session, and post-work for a single module and session in their course
  (c) Fall 2020 Syllabi were due to the Curriculum Office by 31 July 2020
  (d) Each instructor was given an opportunity to conduct a dry-run of their course module (the pre-work and asynchronous instruction, the aligned synchronous session with application-based learning activities, and the associated post-work) in order to identify the strengths and areas for improvement in the process. Student volunteers were assigned to different courses, completed the asynchronous work for the assigned course, attended the aligned synchronous session, then provided valuable feedback to the course instructors regarding content organization, access to course materials, and overall experience in the new delivery format. Instructors used the feedback to revise the course prior to the beginning of the Fall semester
  (e) Lastly, course pages in the LMS were to be completed with syllabus, modules, assignments, etc. one week prior to the first day of classes.

### 2.3. The Student Experience

The newly-implemented instructional format across the curriculum was also new to the students. Although the students had experienced a handful of courses in the flipped classroom, team-based learning (TBL) and asynchronous/synchronous formats, there was a steep learning curve in adapting to all courses being delivered in an asynchronous/synchronous format while living with all other pandemic-related life changes. In addition to a different teaching/learning structure, the semester was shortened by 3 weeks across all schools in order to prevent COVID19 infection rate increases after the Thanksgiving holiday, further increasing the pressure on faculty and students to complete courses in a 13-week time frame. A mid-semester survey was developed and distributed electronically to all students during week 5 of the Fall semester. The survey addressed the following factors: organization of course content, consistency of course modules with syllabus, timely access to asynchronous course material in the LMS, pace of the course, engagement during synchronous sessions, access to faculty, and opportunities for application of asynchronous material.

Although the return on investment cannot yet be addressed, we continue to evaluate the factors that identify specific successes and monitor the factors that we can change. During this process we evaluated student satisfaction, faculty satisfaction, student engagement and how they participate in learning activities, and performance in IPPEs and APPEs. Any instructional or operational “lessons learned” were shared in faculty forums so that changes could be made for the Spring courses. For example, we identified that one type of polling software was preferred by students over others and resulted in increased engagement during synchronous sessions. Another example is that a change in exam processes was implemented due to four academic integrity violation reports this Fall versus 1 in the previous year. The school invested a great deal of effort in student, faculty and staff wellness. The Assistant Dean of Equity, Diversity and Inclusion added a significant amount of wellness activities and forums over the course of 9 months. We continue to monitor student overall wellness, their level and frequency of engagement in wellness activities. We continue to monitor these factors because in this iterative process there is no beginning or an end, only ongoing revisions of a process that lead to continued improvements.

### 2.4. Our Digital Transformation Process Moving Forward

The launching of the Fall 2020 semester was just the beginning of our journey. As the semester progressed, we received feedback from our faculty and learners leading to adjustments in the curriculum schedule for the Spring 2021 semester. Frequent meetings with our course coordinators continue to provide opportunities to encourage further experimentation, collaboration and new ways of thinking. Administratively, we have had some success addressing policies that may have hindered faculty members willingness to experiment with their teaching. Our university has allowed faculty to choose (or not choose) to delay promotion reviews for up to a year. Furthermore, due to the disruption and change in focus, each aspect of the evaluation will be based on a three-year trajectory. Thus, creating the conditions for our faculty to feel safe in experimentation. So, when some efforts are less than successful, we know we have the funding, resources and yet another semester to reframe and try to get it right.

## 3. Discussion

It is well documented that change in any organization is a complex task not free from challenges. Most digital leadership concepts and training are generally aimed at presidents and chief executives of organizations [37]. However, in our recent experience in implementing this change, it has become evident that leadership does not always come from the very top. In our case, it wasn’t the university administration that encouraged, planned, resourced or held faculty accountable to new educational systems, but it was the school leadership who had the vision and plan for this task and dedicated faculty who carried out the vision. The process of leading change has been well-studied and described in multiple iterations by Kotter. Kotter’s updated 8-step process for leading change includes (1) Create a sense of urgency, (2) Build a guiding coalition, (3) Form strategic vision and initiatives, (4) Enlist a volunteer army, (5) Enable action by removing barriers, (6) Generate short term wins, (7) Sustain acceleration, and (8) Institute change [38]. The description of our digital transformation closely follows this process. In March 2020, we were like all academic institutions around the world, in a state of urgency doing what was necessary to sustain operations with significant limitations. The sense of urgency, Kotter’s Step 1, was forced upon us all. At USC, we were fortunate to also have just had created vision for change. Once it was clear that education was going to change for a longer period of time, there was administration support for what was necessary to adapt, and a group of early adopters who generated short term wins and were willing to think creatively and support their faculty colleagues in the process. According to Kotter’s steps of change, the tactical changes we had made in March 2020 put us halfway along in the overall process with results of short-term wins, but we needed to be strategic in order to implement a sustainable and effective long-term solution.

It should be said that digital transformation is not change for the sake of change, or to throw away everything from the past. Digital transformation of education needs to be done cautiously in order to preserve learners’ rich relationships with faculty and classmates, the sense of membership and accountability to the learning community as well as substantive feedback from that community. We know that passive viewership, poor structure without scaffolding, the absence of personalized feedback and force fitting services intended for residential students don’t work, therefore it is imperative to the education of present and future generations that we collectively embrace change in education with creativity and a growth mindset of our own [39]. Practicing agility and adaptability in our education systems will promote a culture of creative educational practices and propel delivery of advanced professional curricula into the new virtual era.

One can say that the digitization of our educational process created by the COVID pandemic was like cramming for an exam. It was a process that required tireless work in a short period of time, with faculty and staff making personal sacrifices almost around the clock, shifting to remote learning to achieve a short-term goal. In contrast, digital transformation of education is a strategic shift, much like the science of effective learning and retention to achieve deep learning [40]. We need to make strategic decisions to adopt a new way of life, be disciplined and focused in our practice, be open to learn from each iteration, and finally be able to thrive in the future we create.
